# Three-Year Chronic Consumption of Low-Carbohydrate Diet Impairs Exercise Performance and Has a Small Unfavorable Effect on Lipid Profile in Middle-Aged Men

**DOI:** 10.3390/nu10121914

**Published:** 2018-12-04

**Authors:** Karol Pilis, Anna Pilis, Krzysztof Stec, Wiesław Pilis, Józef Langfort, Sławomir Letkiewicz, Cezary Michalski, Miłosz Czuba, Michał Zych, Małgorzata Chalimoniuk

**Affiliations:** 1 Institute of Physical Education, Tourism and Physiotherapy, Jan Dlugosz University, 42-200 Czestochowa, Poland; k.pilis@ajd.czest.pl (K.P.); a.pilis@ajd.czest.pl (A.P.); krisstec@gmail.com (K.S.); w.pilis@ajd.czest.pl (W.P.); letkiewicz1@tlen.pl (S.L.); c.michalski@ajd.czest.pl (C.M.); mzych87@interia.pl (M.Z.); 2 Department of Physiotherapy, Opole Medical School, 45-060 Opole, Poland; 3 Department of Sports Training, the Jerzy Kukuczka Academy of Physical Education in Katowice, Faculty of Physical Education, 44-100 Katowice, Poland; langfort@imdik.pan.pl; 4 Department of Kinesiology, Institute of Sport, 01-982 Warsaw, Poland; 5 Faculty of Medicine and Health Sciences, University of Zielona Góra, 65-417 Zielona Góra, Poland; 6 Department of Tourism and Health in Biala Podlaska, Józef Piłsudski University of Physical Education in Warsaw, 00-968 Warsaw, Poland; mchalim@yahoo.com

**Keywords:** diets, carbohydrate restriction, metabolism, men, cardiovascular disease

## Abstract

The objective of this research was to determine whether chronic (average 3.58 ± 1.56 years) deliberate adherence to low carbohydrate diets (LCDs) is associated with selected markers of metabolism, risk factors of cardiovascular disease (CVD), body mass and physical performance in apparently healthy middle-aged men (*n* = 12). The control group comprised age, body mass and height matched men using mixed diets (MDs). The diets used were registered for 7 days and analyzed in terms of the energy, carbohydrate, fat and protein contents. It was found that the diets used were isoenergetic, yet varied considerably in carbohydrate and fat content. The LCDs significantly intensified the ketogenesis process, increased resting blood total cholesterol (TC), high-density lipoprotein cholesterol (HDL-C), and heart rate, (HR) and decreased respiratory exchange ratio (RER) in relation to MD subjects. An exercise trial revealed significant impairment of exercise in subjects following the LCDs. The results showed that in the case where the subjects of two investigated groups did not differ in their somatic variables, long-term adherence to the LCDs was associated with substantially reduced exercise performance in apparently healthy subjects, along with an association with a small unfavorable effect on their lipid profile.

## 1. Introduction

Physical activity and the type of diet are important factors regulating the metabolism of the body and thus affecting the state of human health. In the search for a type of diet that could improve human health, some advantages of low-carbohydrate diets (LCDs) and especially its variety called the Atkins diet were noted. In Poland, the Atkins diet was modified by Dr Kwaśniewski [1], who called it an “optimal diet”. This diet maintains the proportion of proteins/fat/carbohydrate in the range of 1:2.5–3.5:0.5, whereas it does not restrict total energy intake. Moreover, this diet eliminates the following foods: honey, jam, sweets, sucrose, bread, white rice, beans, starch, potatoes and sweetened drinks.

LCDs are similar to high-fat diets (HFDs) and are a broad category lacking an objective definition. The carbohydrate amount of 45–65% of the total daily energy intake is suggested as appropriate for adults [2]. Diets with intakes below 45% can be viewed as LCDs. These diets have also been defined as having an upper limit of 40% of total daily energy from carbohydrate [3,4] or having less than 200 g of this dietary ingredient (3). A more restricted term for LCD suggests specifying non-ketogenic LCD, which contains 50–150 g of CHO, because pure ketogenic diets have a maximum of ~50 g or ~10% of total daily energy from this ingredient [5]. This diet maintains protein at a moderate level (1.2–1.5 g/kg/day) with predominance of energy intake from fat (~60 to 80% or more) [6]. In the light of these criteria, the Atkins and Kwaśniewski diets can be partially included in the ketogenic diets, which ought to be confirmed by a significant increase in the concentration of β-hydroxybutyrate in blood [7,8].

Most studies connected with LCDs have been performed on sick individuals, because this diet is believed to improve the lipid profile and glycemic control [9,10,11]. Moreover, LCDs with energy restriction are used by obese or overweight subjects in weight loss programs [12,13]. Soenen et al. [14] demonstrated that the higher protein content of LCDs rather than lower carbohydrate amount in this diet was the crucial factor in greater weight loss during applied hypocaloric nutrition. It is worth pointing out that non-calorically restricted ketogenic diets have also led to body fat and/or body weight reduction [15,16]. However, this effect seems to occur as a spontaneous energy intake reduction, which could increase satiety through suppression of ghrelin production [17]. Furthermore, it was found that a ketogenic diet does not increase cardiac risk factors in hypercholesterolemic people [18], and an LCD with energy intake coming from CHO below 20% also showed no negative cardiovascular risk in obese individuals with type 2 diabetes [19]. Another field of interest concerning a beneficial impact of a ketogenic diet is its influence on athletes’ exercise performance [20,21,22]. However, in contrast to the proposed benefits of fat adaptation for physical performance, another study showed increased fat oxidation but high-intensity work output was impaired [15,23,24].

High fat intake leads the body to become fat-adapted or keto-adapted. However, so far, long- term studies lasting only up to 2 years have been performed in individuals affected by so-called non-communicable diseases. The main objective of this observational retrospective study was to determine whether 3 years’ adherence to an LCD is associated with metabolic, cardiovascular, plasma lipids and somatic variables of middle-aged men who began to consume this diet as healthy subjects, being convinced of its beneficial protection against susceptibility to disease. Their counterparts were volunteers with matched age, weight and height who used mixed diets (MDs). The intervention part of this study consisted of graded exercise performed to individual maximum load. Our assessment was focused on a survey of risk factors of metabolic and cardiovascular diseases, plasma lipids as well as somatic and exercise capacity variables.

## 2. Material and methods

### 2.1. Participants

Fifteen apparently healthy men who self-reported adherence to LCD for at least 3 years volunteered to participate in this study. All participants had current medical examinations, without any contraindications to performing exhaustive exercise. After medical examination performed by a general practitioner 3 subjects were eliminated from the study, so 12 participants took part in this observation. They declared that they had never engaged in regular physical activity of moderate to vigorous intensity. However, we did not control the varied levels of fitness among the participants. The LCDs subjects were members of local supporters belonging to a nongovernmental society called the “All-Polish National Association of Optimal Brotherhoods”. They declared that they had maintained an LCD for at least 3 years (mean = 4.58 ± 1.1, min = 3, max = 6.5 years). The control group for subjects who applied an LCD comprised 12 volunteers with matched age, weight and height who used an MD all the time. All of the study participants were informed of the objective of the experiment and the accompanying risks. Volunteers provided their written, voluntary, informed consent before participation.

Inclusion criteria were: (1) LCD for at least 3 years; (2) age 40–60 years; (3) BMI 20–29.9 kg/m^2^; (4) body mass 50–90 kg; (5) the lack of chronic diseases; (6) systolic blood pressure 100–140 mmHg and diastolic blood pressure 60–90 mmHg. The study excluded participants with (1) using drugs, drinking alcohol and smoking; (2) hypertension; (3) prematurely stopped exercise test.

The research project was conducted according to the Helsinki Declaration and was approved by the Ethics Committee for Scientific Research at the Jerzy Kukuczka Academy of Physical Education in Katowice, Poland.

### 2.2. Experimental Design

All healthy participants came to the laboratory in the morning, between 8:00 and 9:30 AM, after an overnight fast and abstention from alcohol, medications and exercise for 2 days. In the first stage of the study age and basal somatic data (body height—BH, body mass—BM, body fat—BF, free fat mass—FFM, total body water—TBW and body mass index—BMI) were recorded. Variables were estimated by bioelectrical impedance analysis using the Tanita Body Fat Analyzer TBF 300A (Tanita, Amsterdam, Netherlands). All participants provided to the laboratory a 7-day dietary enrollment recorded with a 24-h dietary recall form completed to assess their habitual daily energy and nutrient intakes. All nutrient data were analyzed using the National Food and Nutrition Institute computer database (Dietus, BUI INFIT, Warsaw, Poland).

Before the incremental exercise test the rest heart rate (HR) and blood pressure (BP) were measured (Oxycon-Alpha ER 900, Jaeger, Hoechberg, Germany). Also, blood samples from the antecubital vein were drawn for determination of concentrations of the following biochemical variables: glucose (G), lactate (LA), uric acid (UA), β-hydroxybutyrate (β-HB), free fatty acids (FFA), total cholesterol (TC), high-density lipoprotein cholesterol (HDL-C), triacylglycerols (TG), and immunoreactive insulin (IRI). Low-density lipoprotein cholesterol (LDL-C) concentration was calculated.

Then after a 10-min rest the blood pressure (systolic—SBP and diastolic—DBP), HR, oxygen uptake (VO_2_), carbon dioxide excretion (VCO_2_) and respiratory exchange ratio (RER) were recorded. On the basis of the obtained data, the following physiological variables were calculated: mean arterial pressure (MAP), pulse pressure (PP), and rate pulse pressure product (RPP). After this time, the subjects from both groups sat on the cycloergometer (ER 900, Jaeger, Hoechberg, Germany) and started work at a rate of 60 revolutions per minute, beginning from 0 W. The load was increased by 30 W to an individual maximum fatigue, maintaining each work stage for 3 min. At maximal load, the above-mentioned physiological variables were recorded and calculated. At each submaximal load HR, VO_2_, VCO_2_ and RER were also recorded. Expired air was analyzed at rest and during an incremental test using the cycloergometer and an Oxycon-Alpha quick gas analyzer (Jaeger, Hoechberg, Germany).

Biochemical analyses were determined in fasting blood samples, which were collected in heparin- or EDTA-treated tubes. Serum and blood plasma were separated, and were assayed immediately for determination of glucose (G), uric acid (UA) and LA concentrations using diagnostic kits: GL 2623 and UA 230 from Randox and BioMérieux Laboratories Ltd., respectively (Spectrophotometer UV-VIS 1202, Shimadzu. Blood samples for determination of β-HB were rapidly deproteinized by addition of 0.6 N perchloric acid. Protein free supernatants, part of plasma and serum were stored at −80 °C and analyzed using a commercial (RANBUT) Randox kit. Plasma TC, HDL-C, TG and serum FFA were determined with enzymatic methods using commercial Randox kids (CH 200, CH 203, TR 1697, and FA 115, respectively) (Spectrophotometer UV-VIS 1202, Kyoto, Shimadzu). Level of low-density lipoprotein cholesterol (LDL) was calculated using the Friedewald formula [25]. Risk for cardiovascular disease (CVD) was evaluated by calculation of the ratios of TC/HDL-C (R_1_), LDL-C/HDL-C (R_2_), and TG/HDL-C (R_3_).

Serum IRI concentration was quantified by the electrochemiluminescence method using an Elecsys 1010 analyzer (Roche Diagnostics, Mannheim, Germany). From the ratio of multiplication fasting G (millimole per liter) and IRI concentrations (milliunits per liter) divided by 22.5 we calculated the homeostasis model assessment (HOMA_IR_) [26]. Both indices, i.e., TG/HDL-C and HOMA_IR_, were applied as surrogate measures of insulin resistance [26].

### 2.3. Statistical Analyses

Results were reported as mean values ± SD. The Shapiro-Wilk test was performed to verify the distribution of the variables. The nonparametric Mann-Whitney *U* test and two-way ANOVA test (for repeated measures) with post hoc Bonferroni test were used to assess the differences between variables of both investigated groups. The tests were set with a confidence interval of 95% and differences with *p* < 0.05 were accepted as statistically significant. The statistical analyses were conducted with STATISTICA 12.0 software version (StatSoft, Krakow, Poland).

## 3. Results

Subjects who participated in the study reported adherence to the high-fat LCD for more than 3 years (mean = 4.58 ± 1.1, min = 3, max = 6.5 years). Basic somatic characteristics for the two study groups are presented in Table 1. Age of both groups was similar and none of the somatic data differed significantly.

It was found that average energy intake was limited to approximately 2075 kcal/day in the LCDs subjects and 1870 kcal/day in the MDs group, and did not differ significantly (Table 2).

Average CHO intake was limited approximately to 118 g/day (23% total daily energy intake) in the LCDs group and was lower than in the MDs subjects—229 g/day (49% total daily energy intake)—*p* < 0.001. Moreover, the average fat intake was approximately 150 g/day (65% total daily energy intake) in LCDs participants and 77 g/day (37% total daily energy intake) in MDs men, whereas protein intake was a little lower than the recommended dietary allowance (it ranged from approximately 0.89 g/kg _bm_ to 0.93 g/kg _bm_ and did not differ significantly between the groups) [27].

Mean values for FFA, β-HB, G, UA, IRI and LA were measured in blood samples and are given in Table 3. FFA (in all samples), β-HB (in LCDs men), G (in LCDs group at maximal exercise) markedly exceeded upper borderline levels. Kind of applied diet (F = 3.45, *p* < 0.05) and maximal physical effort (F = 3.72, *p* < 0.05) were associated with increased plasma FFA concentration and post hoc analysis showed that this level in the LCDs group at maximal exercise was significantly higher than in the MDs group (Cohen’s d = 1.52) and at rest (Cohen’s d = 0.44) in the first abovementioned group (*p* < 0.01). LCDs were associated with increased β-HB plasma concentration (F = 20.4, *p* < 0.001) and it was significantly higher in the LCDs group than in the MDs group at rest (Cohen’s d = 3.2) and maximal exercise (*p* < 0.01; Cohen’s d = 0.32). Moreover increased blood LA concentration was associated with applied physical effort (F = 104.60, *p* < 0.001). LA concentration in blood at maximal exercise level was significantly higher than at rest in both investigated groups (*p* < 0.001; LCDs—Cohen’s d = 2.98; MDs—Cohen’s d = 3.8).

Table 4 presents the levels for principal biomarkers of CVD risk associated with traditional lipid parameters (TC, HDL-C, LDL-C, TG, TC/HDL-C, LDL-C/HDL-C, TG/HDL-C), HOMA_IR_, at rest and maximal intensity exercise. TC levels in LCDs subjects at rest and maximal exercise, and in MDs men under a maximal effort; HDL-C concentration in LCD group at maximal exercise; LDL-C serum concentration in LCDs men at rest and maximal exercise and in the MDs group at rest, and TC/HDL-C ratio calculated at rest in the LCDs group exceeded the upper reference limits.

The kind of applied diet was associated with increased TC concentration (F = 21.8, *p* < 0.001) and it was higher at rest (Cohen’s d = 1.84) and maximal exercise (Cohen’s d = 1.73) in LCDs subjects than in MDs participants. Physical effort was also associated with increased serum concentration of this variable (F = 17.87, *p* < 0.001), where this level in LCDs subjects at maximal exercise was higher than at rest (*p* < 0.05; Cohen’s d = 0.82). Analysis of variance indicates the association between diet (F = 8.95, *p* < 0.01) and physical exercise (F = 16.99, *p* < 0.001) with the level of HDL-C. It was higher (*p* < 0.05) at rest in LCDs subjects than in the MDs group (Cohen’s d = 0.48), and exercise level in LCDs subjects was significantly higher in comparison with resting values (*p* < 0.01; Cohen’s d=0.4). Moreover, used diets were associated with increased LDL-C serum concentration (F = 20.4, *p* < 0.001), and at maximal exercise this level was higher in LCDs men than in MDs subjects (*p* < 0.01; Cohen’s d = 1.74).

Maximal workload during the incremental test (WR_max_) was significantly lower in the LCDs group (145.00 ± 28.1 W) than in the MDs group (175.00 ± 35.8 W), and total work (TW) reached during the ergocycle test (reached the levels-USUNĄC) was 78.3 ± 29.4 kJ in the LCDs group and 112.2 ± 42.2 kJ in MDs subjects, which were significantly different (*p* < 0.05).

Participants had a normal resting reference range of HR (<90 bpm), SBP (<130 mm Hg), DBP (<85 mm Hg, except MD group), MAP (<105 mmHg), PP (<63 mmHg) and RPP (<15 bpm × mmHg), whereas these parameters reached individually different levels during maximal effort. Two-way analysis of variance with repeated measures indicated a significant influence of physical effort on HR (F = 498.8, *p* < 0.001), SBP (F = 142.01, *p* < 0.001), MAP (F = 46.16, *p* < 0.001), PP (F = 148.16, *p* < 0.001), and RPP (F = 412.71, *p* < 0.001). Post hoc analysis also showed (Table 5) that these parameters measured or calculated at rest were significantly lower than at maximal physical effort in both groups (*p* < 0.001).

Mean values of HR and respiratory variables presented in Table 6 recorded at rest and during maximal exercise were within the reference range. The analysis of variance revealed the association between applied diet on HR (F = 9.6, *p* < 0.01), VO_2_ (F = 13.57, *p* < 0.01) and RER (F = 15.75, *p* < 0.001). Post hoc calculations showed that between-group differences were present at rest, 30 W, 60 W, 90 W, 120 W and maximal load, in relation to HR, VO_2_ and RER, respectively. Moreover, significant changes in the above-mentioned variables were observed under the influence of physical effort in HR (F = 216.1, *p* < 0.001), VO_2_ (F = 781.18, *p* < 0.001) and RER (F = 69.77, *p* < 0.01). In post hoc analysis there were observed significantly higher exercise values of HR, VO_2_ and RER in comparison to their resting level in both study groups.

## 4. Discussion

### 4.1. Participants

The main cohort of volunteers of the All-Poland Association of Optimal Brotherhoods comprises ailing individuals of all ages. The main goal of their membership in the association is non-pharmacological support for treatment of obesity, diabetes, cardiovascular risk, or reduction of excessive body weight and the firm belief that, when in compliance with the recommended carbohydrate-deprived diet, should help in reversing pathological changes. However, a small apparently healthy subpopulation of members, prevailingly women, decided to follow LCD guidelines because of the strong conviction of the general prevention of various diseases associated with adherence to this diet. From this group, we selected for participation in the study men who had been on the LCD**s** for at least three years. This criterion is responsible for the relatively small (*n* = 12) number of participants enrolled in the present investigation as well as for the fact that this relatively small cohort does not represent a good cross section of the community. Also a wide spectrum of known short-term harmful effects (for instance fatigue, headache, diarrhea) are directly linked with LCDs both in some pathological states and in athletes who applied such diets during a longer period [28], comments from our participants indicate their high level of satisfaction, improved mood state and enthusiasm for adherence to this diet. The control group was matched only for age, weight and height. This restricts our ability to exclude other confounders. However, these variables are most commonly used to control for confounding, as they are known to have major impacts on outcomes. Moreover, the study design based on observational (epidemiological) retrospective methodology and therefore the results were more prone to different biases, particularly the recall bias. The results of this hypothesis-generating study suggest that persistent consumption of LCDs by sedentary individuals may impair their exercise performance and to a small extent, their lipid profile. Regarding these issues, the literature provides information about consequences and effectiveness of this type of diet almost exclusively related to ailing individuals or well-trained athletes [29,30].

### 4.2. Composition of the Used Diet and Ketogenesis

The analysis of both diets used by participants shows that they were isoenergetic and contained a similar amount of protein. In comparison to the MD, LCDs included a substantially higher amount of fats (65.21 ± 8.93%) and substantially lower quantity of CHO (22.5 ± 7.92%). It means that the LCDs used meets the criteria of the low carbohydrate diets [5]. This is also confirmed by the rate of ketogenesis (β-HB—0.51 ± 0.22 mmol/L), which varied around the upper limit of the range for physiological ketosis [30]. In addition, the protein content in the used LCDs was similar to that in the MD. This lack of difference in the content of protein in both diets confirms a similar concentration of UA in the plasma. This compound reflects the amount of consumed purines, a high content of which occurs in high-protein foods. It worth noting that long-term high levels of UA in the blood plasma lead to a reduction of the glomerular filtrate rate (GFR), which can stimulate chronic kidney disease (CKD) and as a consequence may cause higher risk of CVD [30,31]. This necessitates close monitoring of renal function, especially as this diet has a diuretic effect, which may lead to subclinical dehydration.

When the body is deprived of CHO, two metabolic processes are activated: gluconeogenesis and ketogenesis. This metabolic feature is referred to as nutritional ketosis and continues as long as the body is deprived of CHO. The classic change in blood after use of low carbohydrate diets, which appears already after 2–3 days in healthy individuals, is increased levels of ketone bodies [32]. In the present study, the level of these compounds measured by the concentration of β-HB was about 5 times higher and reached 0.51 ± 0.22 mmol/L at rest and did not change at maximum effort (0.50 ± 0.26 mmol/L) after using the LCDs. The observed level of β-HB in participants using the LCDs indicates that they did not reach concentrations of ketone bodies even for moderate ketosis (2–5 mmol/L), which occurs most frequently during short-term starvation [33]. Since the β-HB to acetoctane ratio in the blood of healthy individuals is 2:1, it should be assumed that the total concentration of ketone compounds in the blood of tested people using the LCDs is about 0.75 mmol/L, and this concentration of ketone compounds is not harmful to health [34]. It is known that the elevated concentration of β-HB in the blood is usually accompanied by a decrease in muscle and liver glycogen content [35], which is in line with reduced LA levels at rest and maximal exercise seen in LCD subjects. Importantly, an elevated level of ketone bodies allows the body to maintain an efficient energy supply even during starvation, because they provide more ATP compared to glucose per gram of substrate [36].

### 4.3. Exercise Capacity and Fat Metabolism

In comparison to the MDs, the LCDs were associated with a reduction in maximal workload (WR_max_) and total work (TW) during the incremental test by about 17.14% and 30.2%, respectively. Similarly, recent data from Ferreira et al. [37] showed that high-intensity exercise tolerance, when performed under low versus high CHO availability, was reduced by about 20% in physically active men. These results are also in agreement with one of the first reports exploring this area, conducted by Langfort et al. [38], which revealed a similar decrease of mean anaerobic power during the 30 s Wingate test in sedentary individuals following a 3-day ketogenic diet. Taken together, the aforementioned results support the concept that prolonged adaptation to LCDs does not reverse its negative effect on exercise performance which appeared after a few days in sedentary individuals. Reduced exercise tolerance might be related to a lower rate of glycogenolysis due to the LCD-induced lower muscle glycogen content [39] with simultaneous increased fat oxidation, which consequently led to a reduction in the availability of carbohydrates for ATP resynthesis [40].

When the plasma glucose level is low due to restricted carbohydrate consumption, fat and ketone bodies are the predominant energy source, being easily utilized by the heart, kidneys, brain and skeletal muscles [40,41]. According to data obtained from lean body endurance-trained athletes, it follows that the low-carbohydrate diet causes further increased utilization of fats over values induced by the training process [23]. Our study showed that a prolonged sustained LCDs were associated with a higher rate of fat utilization across all exercise intensities during the incremental test conducted on sedentary subjects in whom the LCDs was not used in combination with a training process. This conclusion follows from the observed lower values of RER accompanied by a significantly higher VO_2_ at the same submaximal exercise loads. These data indicate that the LCDs can have a negative effect on the energy cost of exercise by increasing the oxygen demand. Previous studies also indicated that the LCDs led to a switch of metabolism from CHO to fat oxidation [40,41]. This was confirmed by higher plasma non-esterified fatty acids (NEFA) concentration achieved at the maximum load in the LCDs group than in the MDs group and indicates the greater availability of this substrate for working muscles. Because both muscle uptake of plasma NEFA and its metabolism reach the maximum rate at about 50% VO_2_max in untrained healthy individuals [29], a higher RER value seen at higher exercise intensities in individuals on the LCDs provides indirect evidence for greater intra-muscular triacylglycerol hydrolysis and energy supply due to stimulation of muscle hormone sensitive lipase by contracting muscles [42]. It is well documented that the muscle triacylglycerol concentration is increased by a high-fat diet [43]. One should also pay attention to the evidence obtained by others that the dehydration accompanying the use of the LCDs could also affect the limitation of the exercise capacity of people using the LCDs [44].

### 4.4. Plasma Lipid Profile

Horowitz and Klein [45] as well as Helge et al. [46] claim that higher fat oxidation observed with low CHO availability can also increase circulation of very-low-density lipoprotein-cholesterol (VLDL-C), which is oxidized among peripheral tissues. A similar effect was observed in our participants applying the LCDs, because their TC concentration measured at rest and at maximal exercise bout, rest HDL-C level and LDL-C concentration measured at maximal exercise bout were significantly higher than in their MD counterparts. Our results also suggest that LDL-C may represent an additional source of energy both at rest and during exercise after prolonged adaptation to the LCDs.

It is well known that the adverse changes in the plasma lipid profile are related to the high saturated fat content of the diet [47,48]. Therefore, it can be assumed that the LCDs could have caused adverse changes in the lipid profile of the plasma, as the subjects from this group consumed a lot of beef and pork. Our results indicate that these expected adverse changes in our subjects were at least partially compensated by a significant increase in HDL-C. The above-mentioned change profoundly affected the ratios of TC/HDL-C, LDL-C/HDL-C and TG/HDL-C, but the obtained values for the latter two ratios did not exceed the reference values for healthy subjects while TC/HDL-C fluctuated around the upper recommended value for the healthy population [7]. It is generally accepted that the TC/HDL-C and LDL-C/HDL-C ratios are better indicators of CVD risk than plasma levels of TC or LDL-C [49,50]. In addition to raising the HDL-C level, another beneficial effect of LCDs described by others is low plasma TG concentration [11,18,51]. Our study provides evidence that the same effect occurs in healthy subjects leading a sedentary lifestyle after long-term adherence to the LCDs. It was reported that even a small decrease in TG and increase in HDL-C levels led to diminished CVD risk and decreased cerebrovascular mortality [11,52]. Such a lipid profile suggests that the LCDs used by our participants is not a risk for CVD formation, although it contained ~ 118 g/day of carbohydrates. It is believed that diets with carbohydrate content below 30 g/day significantly improve the blood lipid profile [53]. Elevated concentrations of lipids and blood glucose are independent risk factors for CVD formation [54]. They induce postprandial impairment in vascular endothelial function, which is characterized by increased postprandial hypertriglyceridemia, inflammation and oxidative stress [55]. It worth noting that similar adverse metabolic changes are also induced by high carbohydrate foods, causing dysmetabolism and endothelial dysfunction [56]. Interestingly, Tyldum et al. [57] discerned that a single high-intensity exercise performed about 16 h before eating a high-fat meal protects against endothelial dysfunction despite clear meal-induced lipemia. The physiological consequences of such an experimental paradigm in subjects on prolonged use of LCDs need to be investigated.

### 4.5. Carbohydrate Metabolism, Insulin Sensitivity and Glucose Tolerance

Metabolism of the body during the use of LCDs by metabolic syndrome, pre-diabetes and diabetes patients causes changes which include increased energy gain from the breakdown of fats and ketones and leads to a number of physiological consequences such as weight loss or lower fasting blood glucose, which is known to reduce risk of CVD [58,59]. Interestingly, when using diets with a very low carbohydrate content, neither decreased insulin nor decreased glucose levels were observed, while there was an increased rate of protein hydrolysis [5]. This last process may augment anaplerotic entry of some amino acids into the tricarboxylic acid cycle. Our studies showed no changes in insulin and glucose levels between investigated groups both in resting and exercise conditions. However, it should be noted that the LCDs used by our participants was not restrictive, as it contained as much as ~22.5% CHO, and therefore the rate of carbohydrate oxidation could be similar as in the MD subjects, which is indirectly indicated by the same plasma LA levels [37]. It follows that in healthy people who use an LCD for several years, there are adaptive changes that maintain insulin and glucose levels in the physiological ranges despite reduced carbohydrate content, as confirmed by Peters et al. [60] and Carey et al. [61], and that is why they are protective against CVD.

The results of the present study show that people using a long-term LCDs do not have a glycemic control disorder, which is confirmed by the HOMA_IR_ index and serum insulin concentration [19]. Another indicator that can be used in the assessment of insulin resistance is the ratio of TG/HDL-C [62], which was also lower in both our groups than the upper limit of the reference range, as in the previous study of Grieb et al. [7]. Rewers et al. [63] and Hanley et al. [64] postulate that the deterioration of insulin sensitivity is one of the most important factors for the formation of atherosclerosis. In the light of these data, there is no evidence indicating that the LCDs used by our participants should be considered as a risk factor for the development of CVD. Helge [65] expressed a similar opinion, postulating that neither low carbohydrate content in the diet, a high-fat diet, nor a high-fat diet coupled with physical training can be considered as factors conducive to the development of impaired carbohydrate tolerance. It was shown that even in the absence of weight reduction low-carbohydrate diets led to improved glycemic control [66,67], improving pre-meal and postprandial blood glucose control [68,69], as well as insulin sensitivity [70]. It was also shown that for glycemic control, apart from low-carbohydrate diets, also the use of high- and moderate-intensity physical efforts was effective, while in the case of high intensity exercise was stronger stimulation [71]. There are also views suggesting that a high-fat diet with low carbohydrate content promotes insulin resistance [72,73], but when it is used for a shorter time than 1 week, it improves glucose tolerance in healthy people [74,75]. Our research showed that the applied LCDs and physical exercise did not impair glucose tolerance and insulin sensitivity, and some of their indicators showed a tendency to improve these variables.

### 4.6. Circulatory System

For many years, a high-fat diet was considered to elicit an extended cardiovascular risk profile [76]. In contrast, recent studies of carbohydrate-restricted diets have shown improvements in markers of cardiovascular health in adults and revealed no negative effect on measures of subclinical disease despite dyslipidemia [77,78,79]. The protective efficacy of long-term adherence to the LCDs in our subjects against a cardiovascular risk profile is seen in some plasma lipid measures. Also there were no harmful changes of SBP and DBP, as well as MAP, PP, and RPP, as compared with values of healthy counterparts. However, other observations show that LCDs can be hazardous for the heart and circulatory system [80,81]. A study by Grieb et al. [7] showed that out of 31 apparently healthy individuals permanently using an LCDs over 1 year, 15 people had SBP below 130 mmHg (upper physiological reference board), 8 in the range 130-139 mmHg (high physiological norm) and 8 in the range 140–170 mmHg (moderate hypertension). Therefore, it can be concluded that a long-term LCDs may be ineffective in reducing hypertension or may even increase it. The above-mentioned cohort, in contrast to our class of subjects, consisted of both sexes, with a considerable age range, and their inclusion in the study necessitated only one-year adherence to LCD**s**.

Our data also confirm the lack of LCD-induced adverse changes in the cardiovascular system by means of blood pressure measurements during exercise. The increase in SBP, MAP, PP and RPP in both groups was similar in relation to resting values, which is commonly known and indicates an efficient cardiovascular response to this type of stimulation. However, an increase in resting HR, achieved under submaximal loads in the conditions of use of the LCDs, in relation to the MD group was noted, similarly as in subjects after very short adherence to the LCD in the study of Langfort et al. [82]. The most likely reason for the increase in resting HR is the stimulation of the sympathetic nervous system, which consequently causes increases in plasma norepinephrine and epinephrine levels [83,84] as well as dehydration-induced tachycardia that often occurs with adherence to the LCDs [85,86]. The achievement of maximum HR at lower loads by subjects on the LCDs indicates that work done during physical exertion was accompanied by a higher energy cost as compared to participants on the MD. Such a diet modification led to the maximum oxygen uptake with a lower exercise load and completion of work at this lower intensity. Also Ferreira et al. [38] observed that the lower availability of carbohydrates in the diet did not change the resting and maximal oxygen uptake in relation to people not using carbohydrate restriction. However, the time of performing the submaximal cycloergometric effort significantly decreased, as in this study.

### 4.7. LCD Impact on Body Mass

The subjects of both groups did not differ in body mass or composition, possibly because of the inclusion procedure for the control group, which complied with age, height and weight matched criteria. This procedure revealed that the diets applied by both groups were isoenergetic. Surprisingly, the increased fat oxidation occurring in the LCDs subjects did not change the content of their body fat in comparison to individuals on the MDs. Perhaps the used LCD**s** contained too much CHO (~118 g/day) and the effect of increased fat oxidation was too small and ineffective in generating somatic changes. The results of most studies indicate that only diets with a carbohydrate content below 30 g/day had a significant effect on the reduction of fat mass in the body [87,88] and most often this reduction is connected with visceral adiposity [89,90].

It is also suggested that the weight reduction elicited by a diet with different carbohydrate content depends on the degree of insulin resistance of the subjects. Cornier et al. [91] showed that greater weight loss occurs in people with normal insulin sensitivity after using hypoenergetic diets with a high carbohydrate content in relation to the hypoenergetic diet with low carbohydrate content. The opposite effect occurred in people with insulin resistance, i.e., greater weight loss occurred after a low-carbohydrate hypocaloric diet. In our study, no insulin resistance was observed, and therefore the influence of this factor on the body mass should be neglected.

### 4.8. Limitations of the Study

Given that our study is based on retrospective observational data, the results we obtained are prone to bias, particularly recall bias, and confounding. These results should therefore be treated as hypothesis-generating. The second limitation of our study is the small number of subjects, who do not represent a good cross section of the community. The third limitation is the use of the 7-day dietary recall method. This may not precisely reflect the composition of the diet of the participants over the previous three years.

## 5. Conclusions

By purposeful selection of a control group, it was possible to eliminate some confounding factors of somatic differences, weight reduction or non-isocaloric diets, which other authors did not avoid because of their experimental paradigm. Our study suggests that the LCDs may impaired exercise performance in apparently healthy men who applied this diet for at least 3 years. However, this acid long-term use of LCD**s** induces physiological ketosis, maintains proper glycemic control and entails favorable somatic profiles. Moreover, this diet is associated with a small unfavorable effect on lipid profile in middle-aged men, and elevated HR at rest and during exercise. It should be pointed out that a single bout of high intensity does not greatly affect these variables.

## Figures and Tables

**Table 1:** Somatic characteristics of participants in the low carbohydrate diets (LCDs) and mixed diets (MDs) groups.

Values	MDs, *n* = 12	LCDs, *n* = 12	Significance (*p*)
Age [years]	50.75 ± 6.81	50.17 ± 8.81	NS
Body height [cm]	172.83 ± 4.6	170.58 ± 5.38	NS
Body mass [kg]	69.98 ± 5.28	70.19 ± 11.49	NS
Body fat [%]	18.74 ± 3.58	20.13 ± 5.82	NS
Body fat [kg]	13.19 ± 2.99	14.70 ± 6.58	NS
Fat free mass [kg]	56.79 ± 4.17	55.46 ± 5.27	NS
Body water [kg]	41.85 ± 2.87	40.62 ± 3.85	NS
BMI [km/m^2^]	23.38 ± 2.11	24.00 ± 2.37	NS

NS—non-significant difference.

**Table 2:** Daily energy and macronutrient intakes calculated from 7-day observation of participants in the low carbohydrate diets (LCDs) and mixed diets (MDs) groups.

Values	MDs, *n* = 12	LCDs, *n* = 12	Significance (*p*)
Energetic value [kcal/day]	1870.86 ± 233.39	2075.14 ± 416.20	NS
Protein [g/day]	64.76 ± 12.46	62.54 ± 16.17	NS
Protein [%]	14.29 ± 3.35	12.29 ± 2.13	NS
Protein [g/kg]	0.93 ± 0.18	0.89 ± 0.15	NS
Fat [g/day]	76.83 ± 21.62	150.16 ± 32.57	*p* < 0.001
Fat [%]	36.83 ± 8.01	65.21 ± 8.93	*p* < 0.001
Fat [g/kg]	1.10 ± 0.21	2.14 ± 0.20	*p* < 0.001
Carbohydrates [g/day]	228.98 ± 47.11	117.51 ± 50.86	*p* < 0.001
Carbohydrates [%]	48.88 ± 8.71	22.50 ± 7.92	*p* < 0.001
Carbohydrates [g/kg]	3.28 ± 0.49	1.66 ± 0.26	*p* < 0.001

NS—non-significant difference.

**Table 3:** Biochemical variables assessed from blood samples of fasting participants in the low carbohydrate diets (LCDs) and mixed diets (MDs) groups.

Values	Rest		Maximal Exercise Bout		Norms at Rest	ANOVA Results
	MDs	LCDs	MDs	LCDs		
FFA (mmol/L)	0.676 ±0.207	0.764 ±0.187 xx	0.675 ±0.185	0.995 ±0.231 ++	0.1–0.5	x: F = 3.45, *p* < 0.05 +: F = 3.72, *p* < 0.05
β-HB (mmol/L)	0.10 ±0.10	0.51 ±0.22 xx	0.11 ±0.05	0.50 ±0.26 xx	<0.120	x: F = 20.4, *p* < 0.001
Glucose (mg/dL)	89.9 5 ± 7.45	99.11 ±12.41	94.68 ±13.51	109.98 ±11.02	70–105	NS
UA (mmol/L)	5.34 ±1.50	5.85 ±2.08	5.30 ±1.69	6.11 ±2.07	<7.00	NS
Insulin (mU/L)	6.65 ±2.80	6.78 ±2.97	6.11 ±2.72	6.06 ±2.81	2.6–24.9	NS
Lactate (mmol/L)	1.79 ±0.31	1.43 ±0.17	6.46 ±1.71 ++	5.31 ±1.83 ++	<2.00	+: F = 104.60, *p* < 0.001

x—MDs vs. LCDs; x—*p* < 0.05; xx—*p* < 0.01; +—Rest vs Maximal exercise bout; +—*p* < 0.05; ++—*p* < 0.01; NS—non-significant difference.

**Table 4:** Biomarkers of cardiovascular disease (CVD) risk estimated in fasting blood samples of participants in the low carbohydrate diets (LCDs) and mixed diets (MDs) groups.

Values	Rest		Maximal Exercise Bout		Norms at Rest	ANOVA Results
	MDs	LCDs	MDs	LCDs		
TC (mg/dL)	197.74 ±24.48	252.82 ±34.36 x	211.99 ±27.15	276.93 ±45.35 x+	130–200	x: F = 21.8, *p* < 0.001 +: F = 17.8, *p* < 0.001
HDL-C (mg/dL)	57.45 ±18.16	65.94 ±16.71 x	60.79 ±16.13	73.13 ±19.13 ++	35–70	x: F = 8.95, *p* < 0.01 +: F = 16.9, *p* < 0.001
LDL-C (mg/dL)	136.24 ±25.42	172.78 ±42.15	122.39 ±34.55	189.45 ±41.17 xx	<135	x: F = 20.4, *p* < 0.001
TG (mg/dL)	93.80 ±34.18	89.74 ±19.43	97.57 ±33.14	90.90 ±17.43	<150	NS
R1 = TC/HDL-C	3.45 ±0.84	4.05 ±1.10	3.64 ±0.70	3.98 ±1.02	< 4.0	NS
R2 = LDL-C/HDL-C	2.58 ±0.83	2.82 ±1.15	2.19 ±0.93	2.77 ±0.97	<4.5	NS
R3 = TG/HDL-C	1.85 ±1.12	1.50 ±0.65	1.73 ±0.83	1.35 ±0.49	≤3.5	NS
HOMA _IR_ mU/mmol	1.47 ±0.64	1.47 ±0.82	1.43 ±0.61	1.64 ±0.83	≤2.5	NS

Legend: x—MDs vs. LCDs; x—*p* < 0.05; xx—*p* < 0.01; +—Rest vs Maximal exercise bout; +—*p* < 0.05; ++—*p* < 0.01 TC—total cholesterol, HDL-C—cholesterol HDL, LDL-C—cholesterol LDL, TG—triacylglycerols; NS—non-significant difference.

**Table 5:** Circulatory variables of participants in the low carbohydrate diets (LCDs) and mixed diets (MDs) groups.

Values	Rest		Maximal Exercise Bout		ANOVA Results
	MDs	LCDs	MDs	LCDs	
HR (bpm)	72.00 ± 7.56	85.92 ± 15.12 x	161.33 ± 15.39 +	161.25 ± 17.57 +	x: F = 9.597, *p* = 0.005 +: F = 498.8, *p* < 0.001
SBP (mmHg)	127.50 ± 10.34	129.58 ± 18.52	171.67 ± 22.90 +	171.67 ± 20.71 +	+: F = 142.01, *p* < 0.001
DBP (mmHg)	85.42 ± 8.91	83.33 ± 9.61	89.58 ± 10.10	87.08 ± 11.96	NS
MAP (mmHg)	99.44 ± 8.49	101.25 ± 14.85	116.93 ± 13.36 +	115.29 ± 13.66 +	+: F = 46.16, *p* < 0.001
PP (mmHg)	42.08 ± 8.65	46.25 ± 12.99	82.08 ± 16.98 +	84.33 ± 15.86 +	+: F = 148.16, *p* < 0.001
RPP (bpm x mmHg)	9.22 ± 1.63	11.17 ± 2.76	27.84 ± 5.32 +	27.49 ± 2.61 +	+: F = 412.71, *p* < 0.001

Legend: x—MDs vs. LCDs; x—*p* < 0.05; +—Rest vs Maximal exercise bout; +—*p* < 0.001; NS—non-significant difference.

**Table 6:** Physiological participants’ variables connected with rest and exercise metabolism in the low carbohydrate diets (LCDs) and mixed diets (MDs) groups.

Variables	Group	Rest	Workload [W]					
			30	60	90	120	Max	ANOVA Results
HR [bpm] RER	MDs	72.0 7.56	95.00	108.08	123.75	137.00	161.33	x: F = 9.6, *p* < 0.01
			±9.89 +	±11.94 +	±14.70 +	± 16.70 +	±15.39 +	
	LCDs	85.9 ±15.12 x	107.25	121.58	138.42	153.17	161.25	+: F = 216.1, *p* < 0.001
			±12.56 +x	±13.19 +x	±15.49 +x	±18.77 +	±17.57 +	
VO_2_ [ml/min]	MDs	357.83 ±76.82	898.33	1198.83	1565.67	1955.92	2547.92	x: F = 13.57, *p* < 0.01
			±35.53 +	±106.11 +	±83.95 +	± 89.96 +	±446.49+	
	LCDs	399.42 ±87.47	961.92	1306.75	1724.75	2130.42	2429.92	+: F = 781.18, *p* < 0.001
			±102.57 +	±115.49 +	±152.33 +x	±188.74 +x	±541.97 +	
RER	MDs	0.82 ±0.08	0.86	0.93	1.01	1.06	1.16	x: F = 15.75, *p* < 0.001
			±0.08	±0.08 +	± 0.07 +	±0.06 +	±0.09 +	
	LCDs	0.75 ±0.04 x	0.78	0.85	0.90	0.94	1.01	+: F = 69.77, *p* < 0.01
			±0.05	±0.05 +	±0.05 +x	±0.07 +x	±0.11 +x	

Legend: x—MDs vs LCDs; x—*p* < 0.05; +—Rest vs Maximal exercise bout; +—*p* < 0.001; Upper values of each variable are connected with the MDs group while lower values are connected with the LCDs group; NS—non-significant difference.
